# A Novel Convolutional Neural Network for the Diagnosis and Classification of Rosacea: Usability Study

**DOI:** 10.2196/23415

**Published:** 2021-03-15

**Authors:** Zhixiang Zhao, Che-Ming Wu, Shuping Zhang, Fanping He, Fangfen Liu, Ben Wang, Yingxue Huang, Wei Shi, Dan Jian, Hongfu Xie, Chao-Yuan Yeh, Ji Li

**Affiliations:** 1 Department of Dermatology Xiangya Hospital of Central South University Changsha China; 2 Hunan Key Laboratory of Aging Biology Xiangya Hospital of Central South University Changsha China; 3 aetherAI, Co Ltd Taipei, Taiwan China; 4 National Clinical Research Center for Geriatric Disorders Xiangya Hospital of Central South University Changsha China; 5 Key Laboratory of Organ Injury Aging and Regenerative Medicine of Hunan Province Changsha China

**Keywords:** rosacea, artificial intelligence, convolutional neural networks

## Abstract

**Background:**

Rosacea is a chronic inflammatory disease with variable clinical presentations, including transient flushing, fixed erythema, papules, pustules, and phymatous changes on the central face. Owing to the diversity in the clinical manifestations of rosacea, the lack of objective biochemical examinations, and nonspecificity in histopathological findings, accurate identification of rosacea is a big challenge. Artificial intelligence has emerged as a potential tool in the identification and evaluation of some skin diseases such as melanoma, basal cell carcinoma, and psoriasis.

**Objective:**

The objective of our study was to utilize a convolutional neural network (CNN) to differentiate the clinical photos of patients with rosacea (taken from 3 different angles) from those of patients with other skin diseases such as acne, seborrheic dermatitis, and eczema that could be easily confused with rosacea.

**Methods:**

In this study, 24,736 photos comprising of 18,647 photos of patients with rosacea and 6089 photos of patients with other skin diseases such as acne, facial seborrheic dermatitis, and eczema were included and analyzed by our CNN model based on ResNet-50.

**Results:**

The CNN in our study achieved an overall accuracy and precision of 0.914 and 0.898, with an area under the receiver operating characteristic curve of 0.972 for the detection of rosacea. The accuracy of classifying 3 subtypes of rosacea, that is, erythematotelangiectatic rosacea, papulopustular rosacea, and phymatous rosacea was 83.9%, 74.3%, and 80.0%, respectively. Moreover, the accuracy and precision of our CNN to distinguish rosacea from acne reached 0.931 and 0.893, respectively. For the differentiation between rosacea, seborrheic dermatitis, and eczema, the overall accuracy of our CNN was 0.757 and the precision was 0.667. Finally, by comparing the CNN diagnosis with the diagnoses by dermatologists of different expertise levels, we found that our CNN system is capable of identifying rosacea with a performance superior to that of resident doctors or attending physicians and comparable to that of experienced dermatologists.

**Conclusions:**

The findings of our study showed that by assessing clinical images, the CNN system in our study could identify rosacea with accuracy and precision comparable to that of an experienced dermatologist.

## Introduction

Rosacea is a common chronic inflammatory disease, which mainly affects the convex facial areas such as nose, cheek, chin, and glabella, with estimated prevalence ranging from 2% to 22% worldwide [1,2] and leading to impaired physical appearance, self-abasement, frustration, and poor quality of life in millions of patients with rosacea [3]. The clinical manifestations of rosacea are quite diversified, including flushing, erythema, angiotelectasis, papules, pustules, and phymatous changes [4], which vary largely from patient to patient, and some of these manifestations usually overlap [5]. Besides, the clinical features of rosacea resemble those of a series of facial inflammatory diseases such as acne, seborrheic dermatitis/eczema, and lupus, thereby making the correct recognition of rosacea even more difficult [6]. In addition, the existing clinical diagnostic criteria for rosacea are still debatable and cause confusion in clinical practice [7,8]. Thus, the correct diagnosis of rosacea remains a big challenge for the medical community, and there is a desperate need for a universal reliable diagnostic system for rosacea.

In recent years, with the rapid development of computer science, artificial intelligence has emerged as a promising tool for face recognition, image analysis, and deciphering genomics [9-13]. Among them, the utility of deep convolutional neural networks (CNNs) in medical practice has caught great attention, especially in the field of dermatology [14,15]. Much efforts have been made to apply machine learning in the detection of malignant skin tumors such as melanoma and basal cell carcinoma [16-21]. Early screening and accurate detection of these skin cancers are the premises for timely treatment and would be of great benefit for patients. Furthermore, machine learning can serve as a potential method for identifying other common skin diseases such as psoriasis, atopic dermatitis, and onychomycosis [14,15]. By objectively analyzing and summarizing dermatological images, artificial intelligence can offer clinicians unbiased suggestions for clinical assessment and outcome prediction [14,22], which would effectively narrow the gap between physicians with different educational backgrounds or clinical experience.

In this study, we trained a deep CNN to analyze clinical images (from 3 different angles) of thousands of patients with rosacea versus those of patients with other common diseases, which could be easily confused with rosacea in clinic (eg, acne, facial seborrheic dermatitis, eczema). We aimed to evaluate the ability of our CNN to identify and classify rosacea. We also compared the accuracy and specificity of our CNN in distinguishing rosacea from other skin diseases with those of clinicians with different levels of clinical experiences.

## Methods

The concept of CNN was proposed by Lecun et al [23]. CNN uses various filters to capture features from local regions of an image and shows state-of-the-art performances in many image-based machine learning tasks such as image classification [24], object detection [25], and object segmentation [26]. The common architecture of CNN can be divided into 2 parts: feature extractor and classifier. The feature extractor is composed of stacked convolutional layers and pooling layers. Each convolutional layer contains many filters, which scan the image and do a Hadamard product operation.

After scanning, a filter will generate a 2D matrix called as the feature map (Multimedia Appendix 1). This feature map will progress to an activation function. The most common function is the rectified linear unit, as shown below [23].

y=x, if x≥0

y=0, if x<0

Another common layer in the feature extractor part is the pooling layer. The pooling layer will subsample the feature maps in the height and width domain. It is applied to execute a denoising process. It will sample a value from every 2×2 or 3×3 subregions of the feature maps (Multimedia Appendix 2).

In this way, the pooling layer can reduce redundant information. Reducing the feature map size also decreases the calculation in the following convolutional layers. The sampling strategy in the pooling layers can be done in many ways. In recent years, max pooling is considered as the most efficient strategy in image classification and is used in many CNN architectures. It samples the maximum value from a subregion. The below equation shows how max pooling works.

y = max (X)

The second part of the CNN is the classifier. It is composed of one or many fully connected layers. The feature maps from the feature extractor module will be flattened or downsampled into a 1D vector and fed to the fully connected layers. Each fully connected layer executes a matrix calculation as shown below.

y = h (WX + b)

W means weights, which is a 2D matrix; b means bias, which is a 1D vector; and h (·) is an activation function. The whole CNN will be optimized by backpropagation [27] and gradient descent algorithm [28]. All learnable parameters, including filters, weights, and bias, will be updated during the optimizing procedure.

In our model, we used ResNet-50, which is a variant of CNN, to distinguish rosacea from other facial diseases [29]. ResNet-50 is known as a CNN model with a very “deep” architecture. ResNet-50 overcomes the gradient vanishing problem in case a model becomes deeper and has better generalization than other architectures. The architecture of ResNet-50 is shown below (Multimedia Appendix 3).

The major structure of ResNet-50 is the residual block. ResNet-50 contains 16 residual blocks. Each residual block is composed of 3 convolutional layers. The following figure displays the structure of a residual block (Multimedia Appendix 4).

The first 2 layers in a residual block have the same number of filters. The last layer always has 4 times the number of filters present in the previous layers. The output of a residual block is computed by adding the original input and the output from 3 convolutional layers. After passing 16 blocks, a global average pooling layer samples a value from each feature map by averaging them. Finally, a fully connected layer generates a vector with the output of the global average pooling layer. This vector is the prediction of the model, which contains 2 values and means the scores of rosacea and other facial diseases that might be easily confused with rosacea (such as acne, facial seborrheic dermatitis, and eczema). We applied transfer learning to our model because parameters in the model after pretraining can be considered as a better initialization than initializing parameters randomly at the beginning of the training [30]. Therefore, our model was pretrained with an ImageNet data set, which contains 1 million images, and fine-tuned on our data set [31]. Since our raw images have different resolutions, we should unify them before feeding them into the model. We resized each image to 256 pixels at their shorter side and kept their aspect ratio. After that, we only reserved the central 256×256 region. In this way, we could obtain many 256 pixel×256 pixel images without aspect ratio distortion.

We used facial cropping, rotation, and flipping to augment our data set. For each image, we randomly sampled a 224×224 crop. Then, we rotated the image by 0, 90, 180, or 270 degrees; 25 of the images in a batch were flipped vertically. Further, 25% of the images in the same batch were flipped horizontally. Images may be chosen to flip vertically or horizontally at the same time. We tried to use more affine transformation to augment our data such as more rotation angles, scaling, and shifting. However, we found that too much affine transformation would cause overfitting more easily. Moreover, the performances were also worse than using only a few augmentation methods. We did not consider color augmentation because we believed that the color of patients’ facial skin is one of the keys to determine their disease. Changing the contrast, saturation, and hue would confuse the model.

We optimized our model with mini-batch gradient descent with a momentum of 0.9 and a batch size of 32. Our model was trained for 100 epochs. The initial learning rate was set to 0.0001. If validation loss did not decrease in continuous 10 epochs, the learning rate was divided by 5. The minimum learning rate was not lower than 0.000001. Before training, we randomly split 20% of the data from the training set as the validation set. Further, we used the performance of the validation set to select the best model.

**Figure 1:**
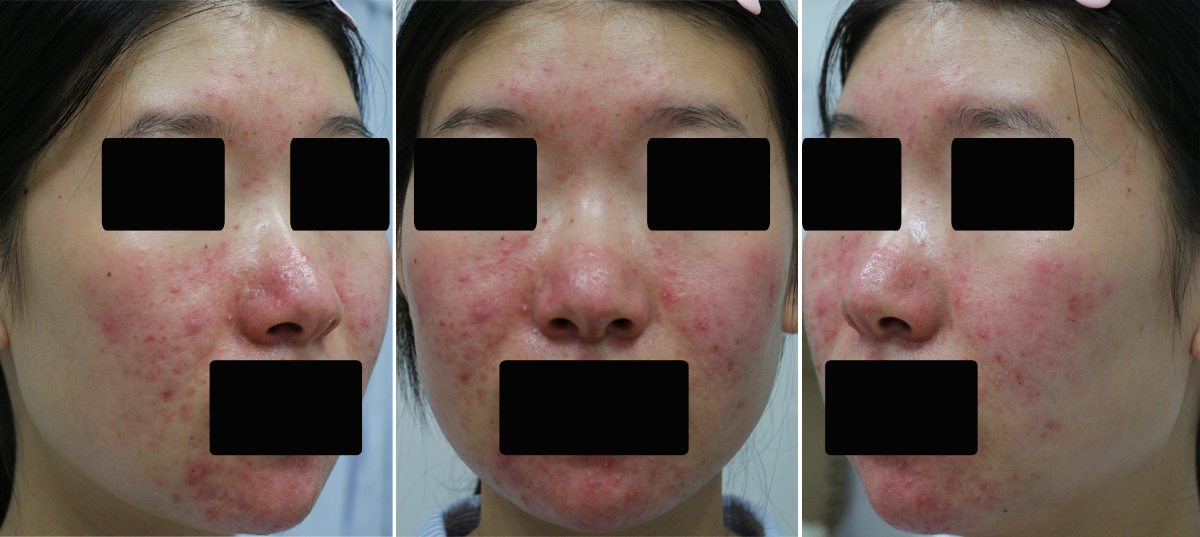
Examples of 3 photos taken from 3 different angles for each patient.

A total of 24,736 photos comprising of 18,647 photos of patients with rosacea and 6089 photos of patients with different skin diseases such as acne, facial seborrheic dermatitis, and eczema were included in our study. The patients in this study gave written informed consent to publish their case details. In order to cover the whole face, the photos of each patient were taken using smartphones (iPhone X and Huawei P20) or digital camera (Canon Rebel 550) from 3 different angles (Figure 1): left face (45 degrees from the left), middle face, and right face (45 degrees from the right).

To build a test set without class imbalance, we did not split the data randomly. Instead, we made sure that each class has the same number of test examples. For each binary classification task, we chose 768 photos from 256 patients (128 patients for rosacea, 128 patients for other skin diseases) as the test set. For rosacea subtype prediction, we chose 576 images from 192 patients (64 patients for each subtype) as the test set. For data analysis, the area under the receiver operating characteristic curve (AUROC) was calculated for each of these curves to quantify the CNN’s performance. A confusion matrix was constructed from the results of the testing images to evaluate the performance.

## Results

### Using Deep CNN to Identify and Classify Rosacea

**Figure 2:**
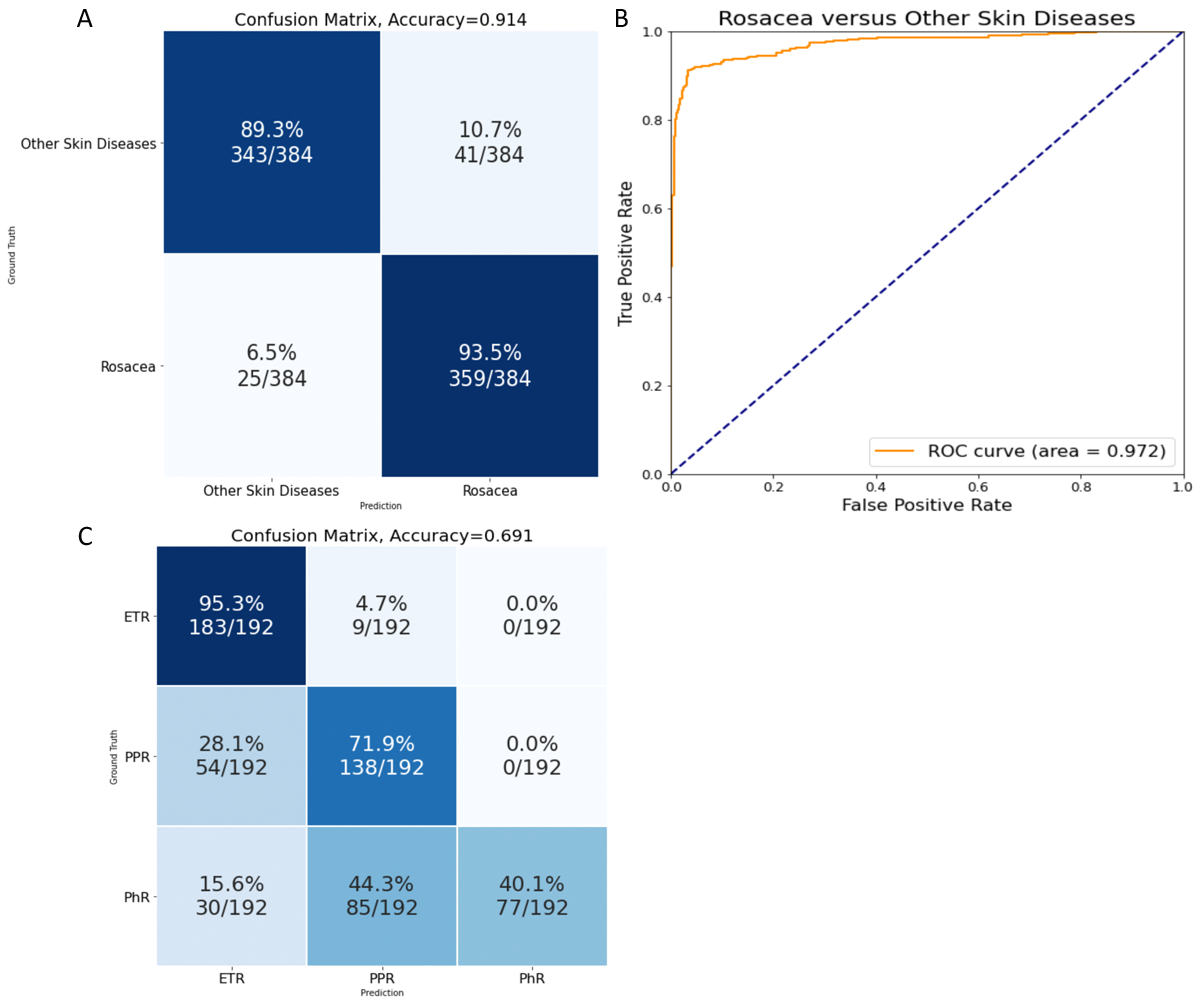
Performance of the convolutional neural network in the identification and classification of rosacea and other skin diseases. A. Confusion matrix showing the accuracy and precision of 0.914 and 0.898, respectively; B. Receiver operating characteristic curve showing that the area under the receiver operating characteristic curve reached 0.972; C. Performance of the convolutional neural network in the classification of subtypes of rosacea. ETR: erythematotelangiectatic rosacea; PhR: phymatous rosacea; PPR: papulopustular rosacea.

First, we tested the ability of CNN to identify rosacea (18,647 images) and other skin diseases, which could be easily confused with rosacea in clinic (6089 images). The latter included acne, facial eczema and seborrheic dermatitis, lupus erythematosus, chronic solar dermatitis, corticosteroid-dependent dermatitis, and lupus miliaris disseminatus faciei. Among them, 23,768 images were used for training and the rest were used for testing. The accuracy and precision pf the CNN for the classification of rosacea against other skin diseases were 0.914 and 0.898, respectively, with an AUROC of 0.972 (Figure 2A and Figure 2B), thereby indicating that CNN was able to identify rosacea effectively and accurately from other skin diseases on the face that might be easily confused with rosacea. Next, we tried to utilize the CNN to further classify the 3 major subtypes of rosacea: erythematotelangiectatic rosacea (ETR), papulopustular rosacea (PPR), and phymatous rosacea (PhR). The accuracy of the CNN to classify one subtype against the others was 83.9%, 74.3%, and 80.0% for ETR, PPR, and PhR, respectively (Figure 2C). To be more specific, 28.1% (54/192) of the patients with PPR were mistakenly recognized as having ETR, while 15.6% (30/192) and 44.3% (85/192) of the patients with PhR were misinterpreted as having ETR and PPR, respectively (Figure 2C).

### Using Deep CNN to Distinguish Rosacea From Acne

**Figure 3:**
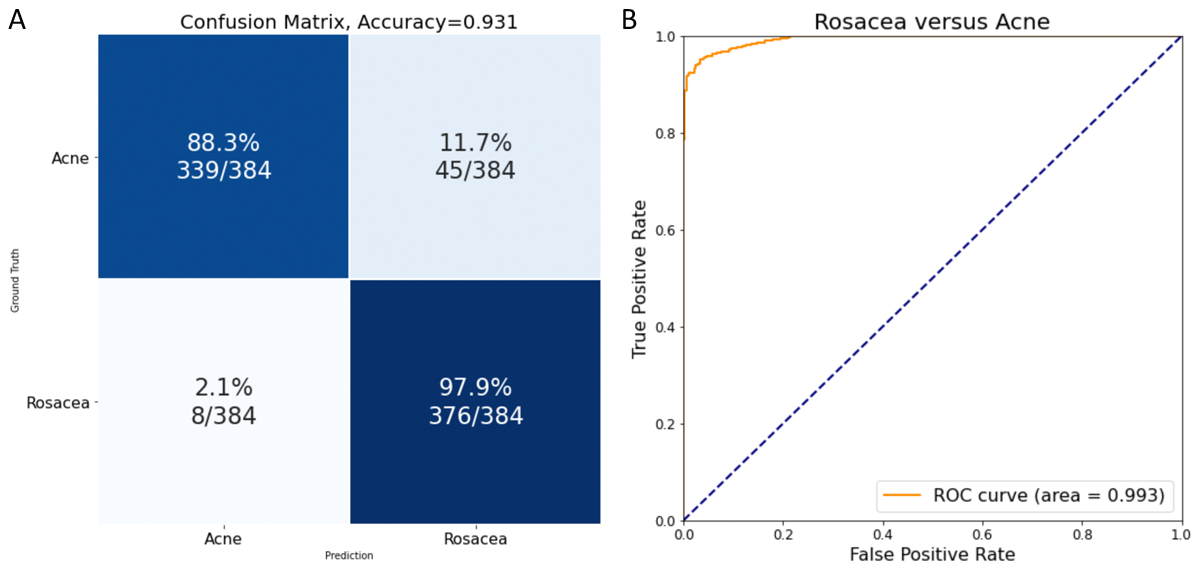
Performance of the convolutional neural network in the identification of rosacea and acne. A. Confusion matrix showing that the accuracy and precision were 0.931 and 0.893, respectively; B. Receiver operating characteristic curve showing that the area under the receiver operating characteristic curve reaches 0.993.

Acne is one of the most important disorders considered in the differential diagnosis of rosacea; therefore, we further proceeded to apply our CNN to distinguish rosacea from acne. The total number of images incorporated into this study was 18,647 for rosacea and 3552 for acne. Among them, 21,431 images were used for training and 768 for testing. The accuracy of this test was 0.931 with a precision of 0.893 (Figure 3A). The AUROC was 0.993 (Figure 3B) and the recall was 0.982. These results demonstrated that our CNN was capable of accurately distinguishing rosacea from acne.

### Using Deep CNN to Distinguish Rosacea From Facial Seborrheic Dermatitis/Eczema

**Figure 4:**
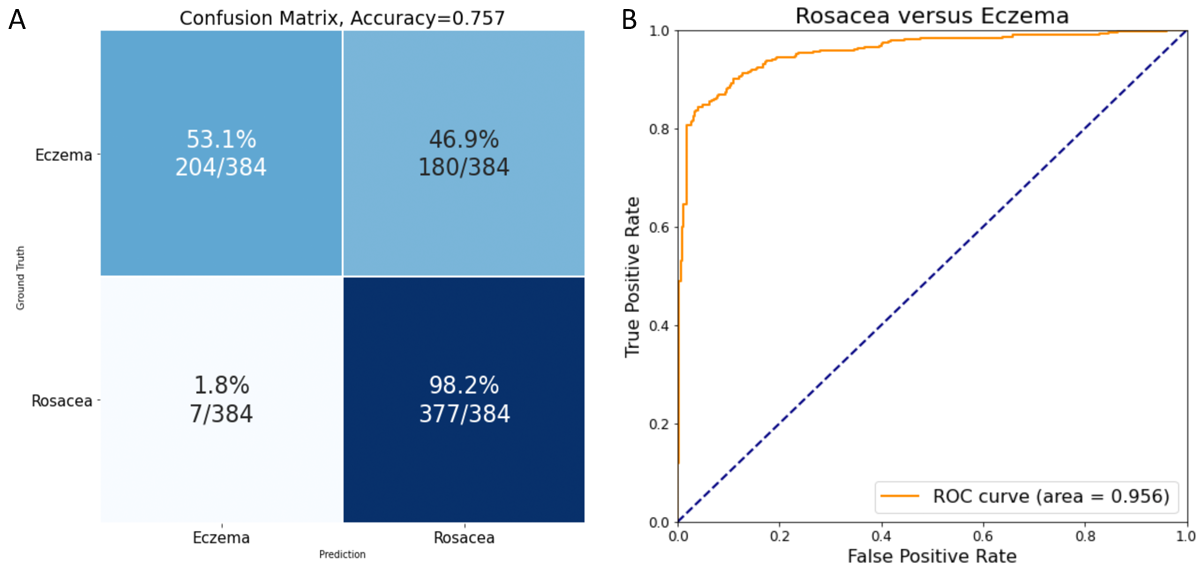
Performance of the convolutional neural network in the identification of rosacea and facial seborrheic dermatitis/eczema. A. Confusion matrix showing that the accuracy and precision were 0.757 and 0.677, respectively; B. Receiver operating characteristic curve showing that the area under the receiver operating characteristic curve reaches 0.956.

Facial seborrheic dermatitis and eczema are other types of facial dermatitis that can be easily misdiagnosed as rosacea in clinical practice. We collected 18,647 images of rosacea and 1896 facial seborrheic dermatitis/eczema images for CNN assessment and identification. After being trained with 19,775 images, the CNN achieved 0.757 for accuracy and 0.677 for precision in the differentiation of rosacea from facial seborrheic dermatitis/eczema on the test set of 768 images (Figure 4A). The overall AUROC of this test was 0.956 (Figure 4B).

### Comparing the Performance of Deep CNN With That of Dermatologists of Different Expertise Levels

**Figure 5:**
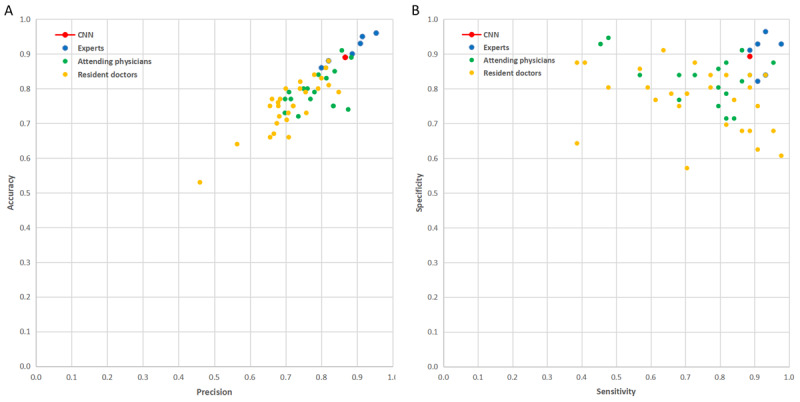
Performance of the convolutional neural network and dermatologists of different expertise levels in the identification of rosacea and other skin diseases. A. Precision and accuracy of the convolutional neural network compared to those of resident doctors, attending physicians, and experts; B. Sensitivity and specificity of the convolutional neural network compared to those of resident doctors, attending physicians, and experts.

We compared the performance of our CNN with that of dermatologists of different expertise levels in the identification of rosacea and other skin diseases. The latter consisted of 6 experts dedicated in the clinical research of rosacea, 19 attending physicians, and 28 resident doctors of dermatology; 44 images of patients with rosacea and 56 images of patients with different skin diseases were used for the test. Compared with our CNN, which achieved an accuracy of 0.890 and precision of 0.867, the overall mean accuracy and precision of the experts were 0.913 (SD 0.040) and 0.881 (SD 0.059), respectively. By contrast, the overall mean accuracy and precision of attending physicians were 0.803 (SD 0.058) and 0.791 (SD 0.063), while those of resident doctors were 0.75 (SD 0.075) and 0.714 (SD 0.081), respectively (Figure 5A and Figure 5B). In summary, these results indicated that the performance of our CNN was significantly superior to that of resident doctors and attending physicians and was comparable to that of experienced dermatologists in the identification of rosacea.

## Discussion

Our study offers a novel CNN that can correctly identify and classify the subtypes of rosacea, and the performance of our CNN is comparable to that of expert dermatologists specialized in the diagnosis and treatment of rosacea. Previous efforts have been made to apply CNN to identify rosacea. However, previous work focused mainly on the development of networks or analysis of images instead of practically applying CNN for the identification of rosacea and differentiating it from other skin diseases or for the classification of subtypes of rosacea [32]. Besides, the number of images for model development was quite limited (less than 100) in the previous studies and the sensitivity or specificity were barely satisfactory [33]. In our work, a vast number of images were incorporated for the training of CNN, and the precision and accuracy of our deep CNN system were 0.914 and 0.898, respectively, for the identification of rosacea among other skin diseases. In addition, in the test for detecting rosacea, our CNN system significantly outperformed the resident doctors and the attending physicians and the performance was comparable to that of experienced dermatologists. Thus, our CNN can serve as a unified detection tool and as a promising adjunct for grassroot health care workers (such as family doctors) to improve their capability in recognizing rosacea and narrow the gap between doctors with different clinical experiences. This, in turn, would be also of great benefit for patients with rosacea since it is not easy for the general public to obtain access to experts for dermatological consultation in daily life.

Traditionally, rosacea is categorized into the following different subtypes: ETR, PPR, PhR, and ocular rosacea [4]. However, the boundary between these subtypes (specially ETR and PPR) is quite obscure and these subtypes may overlap or transform from one subtype to another [7]. The correct classification of the different subtypes in clinical practice has always been a challenge for clinicians. In this study, we tried to utilize our CNN to classify ETR, PPR, and PhR, and the precision was 83.9%, 74.3%, and 80.0%, respectively. To be more specific, 28.1% (54/192) of the patients with PPR were mistakenly recognized as having ETR. One possible explanation for this difference in performance could be the overlapping presentations of ETR and PPR, which is commonly seen in clinic. Moreover, 15.6% (30/192) and 44.3% (85/192) of the patients with PhR were misinterpreted as having ETR and PPR, respectively. Possible reasons for these mistakes could be that the erythema of some patients with ETR and the papules of some patients with PPR were confined to the nasal part, making it difficult to distinguish ETR and PPR from PhR, especially when phymas were not prominent. Nowadays, with the growing understanding of rosacea, this traditional subtype classification has been abrogated by the experts committee for rosacea due to the impractical sorting criteria [34]. The confusions in the classification of the subtypes of rosacea by CNN in our study would in turn support the current consensus of abolishing the impractical classification method.

Generally, the data sources for the interpretation of machine learning varies from digital medical records [35,36], histopathological pictures [37], and clinical photos [38-41] to dermoscopic images [42-50]. The advantage of machine learning over the human eyes is that the CNN is able to objectively record, process, and summarize all the subtle features included in the images of patients with rosacea—even those details that would have been neglected by clinicians otherwise. Each type of data provides unique clinical information for disease diagnosis and at the same time has its own benefits and drawbacks. For example, dermoscopic images are standardized high-resolution pictures, which can clearly present all the microscopic details of the lesion and eliminate the potential interference of image quality and lighting angles with ordinary photos, which makes them especially useful for identifying diseases with specific microscopic patterns (eg, atypical network and irregular dots/globules for melanoma, large blue-grey ovoid nests and spoke-wheel areas for basal cell carcinoma). However, dermoscopic images have a rather limited field of view. For skin diseases such as rosacea, one single dermoscopic image covers only a small proportion of the whole lesion, which hardly represents all the clinical characteristics of the disease comprehensively. In this scenario, clinical photographs taken using the digital camera from 3 different angles covering the whole face (as shown in Figure 1 of our study) would be the preferred image source. Further, clinical photos from different angles also allowed us to analyze areas that were not commonly implicated in rosacea, such as the zygomatic process of the maxilla and the lower mandible. The implication of these areas could be a key point for the differentiation between rosacea and other skin diseases. Additionally, given that each image type has its own limitations, it would be interesting if different types of images (clinical photos, dermoscopic images, and histopathological pictures) were integrated for artificial intelligence assessment at the same time. Future work is encouraged to integrate different types of images providing both microcosmic and macroscopic features of diseases for machine learning to achieve greater performance.

To date, little is known about how exactly deep CNNs analyze, process, and summarize these images and distinguish one disease from another. It remains to be explored what factors (image number, light, background, definition, dimensions) can affect this process. Further efforts are required to investigate the possible ways for improving the accuracy and specificity for the detection of diseases by using artificial intelligence.

In conclusion, our study demonstrated the capability of our deep CNN to identify rosacea and differentiate it from other skin diseases. The performance of our CNN was superior to that of resident doctors and attending physicians and was comparable to that of experienced dermatologists. Our results offer a new potential way for the proper diagnosis of rosacea, thereby indicating that artificial intelligence would be of great help for physicians in the diagnosis of rosacea in clinical practice in the future.

## Appendix

Multimedia Appendix 1 Generation of feature map.

Multimedia Appendix 2 Schema of the denoising process.

Multimedia Appendix 3 The architecture of ResNet-50.

Multimedia Appendix 4 The structure of a residual block.

## Abbreviations

AUROC: area under the receiver operating characteristic curve
CNN: convolutional neural network
ETR: erythematotelangiectatic rosacea
PhR: phymatous rosacea
PPR: papulopustular rosacea
