# Correction: A Novel Convolutional Neural Network for the Diagnosis and Classification of Rosacea: Usability Study

**DOI:** 10.2196/57654

**Published:** 2024-03-08

**Authors:** Zhixiang Zhao, Che-Ming Wu, Shuping Zhang, Fanping He, Fangfen Liu, Ben Wang, Yingxue Huang, Wei Shi, Dan Jian, Hongfu Xie, Chao-Yuan Yeh, Ji Li

**Affiliations:** 1 Department of Dermatology Xiangya Hospital of Central South University Changsha China; 2 Hunan Key Laboratory of Aging Biology Xiangya Hospital of Central South University Changsha China; 3 aetherAI, Co Ltd Taipei, Taiwan China; 4 National Clinical Research Center for Geriatric Disorders Xiangya Hospital of Central South University Changsha China; 5 Key Laboratory of Organ Injury Aging and Regenerative Medicine of Hunan Province Changsha China

In “A Novel Convolutional Neural Network for the Diagnosis and Classification of Rosacea: Usability Study” (JMIR Med Inform 2021;9(3):e23415) the authors made one addition.

An “Acknowledgments” section has been added that reads as follows:

This work was supported by The Educational Science and Planning Project of Hunan Province (XTK20BGD008).

The correction will appear in the online version of the paper on the JMIR Publications website on March 8, 2024, together with the publication of this correction notice. Because this was made after submission to PubMed, PubMed Central, and other full-text repositories, the corrected article has also been resubmitted to those repositories.

