# Supplementary figures and images for: A Novel Convolutional Neural Network for the Diagnosis and Classification of Rosacea: Usability Study
Source: JMIR Med Inform. 2021 Mar 15;9(3):e23415. doi: 10.2196/23415 (PMC8077711; doi:10.2196/23415)

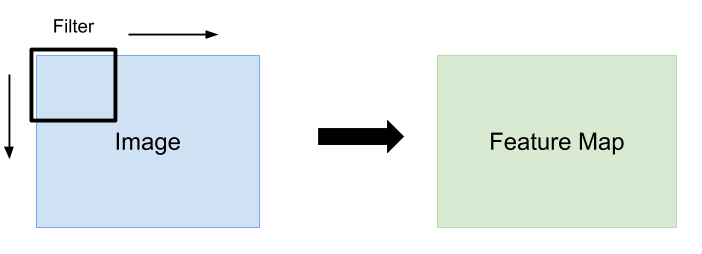

Supplement: Multimedia Appendix 1 [file medinform_v9i3e23415_app1.png]

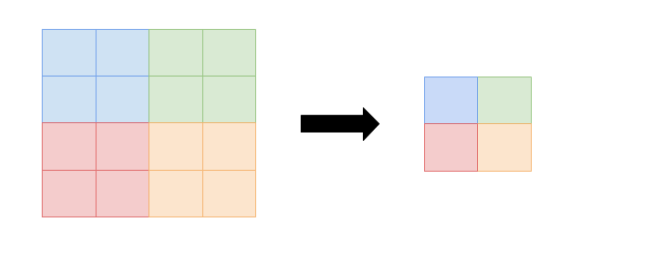

Supplement: Multimedia Appendix 2 [file medinform_v9i3e23415_app2.png]

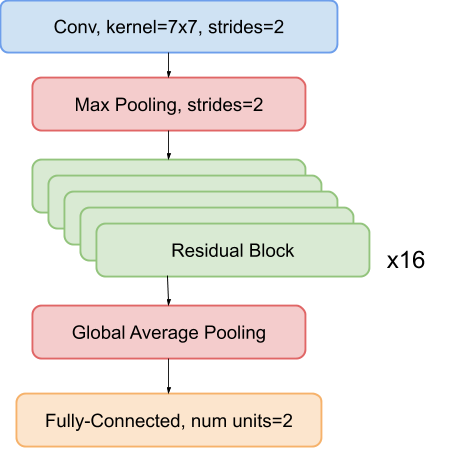

Supplement: Multimedia Appendix 3 [file medinform_v9i3e23415_app3.png]

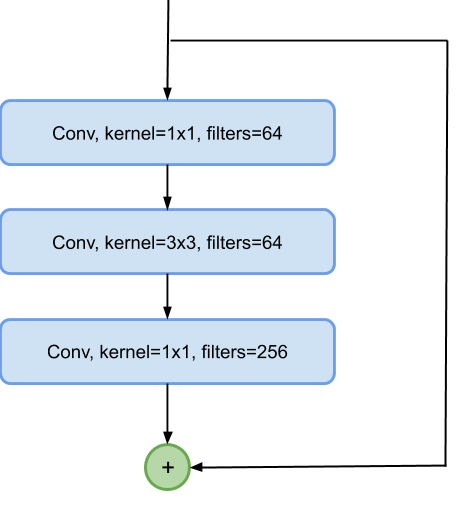

Supplement: Multimedia Appendix 4 [file medinform_v9i3e23415_app4.png]
